# Discriminate the response of Acute Myeloid Leukemia patients to treatment by using proteomics data and Answer Set Programming

**DOI:** 10.1186/s12859-018-2034-4

**Published:** 2018-03-08

**Authors:** Lokmane Chebouba, Bertrand Miannay, Dalila Boughaci, Carito Guziolowski

**Affiliations:** 1 Department of Computer Science, LRIA Laboratory, Electrical Engineering and Computer Science Faculty, University of Science and Technology Houari Boumediene (USTHB), El-Alia BP 32 Bab-Ezzouar, Algiers, 16111 Algeria; 2 LS2N, UMR 6004, École Centrale de Nantes, Nantes, France

**Keywords:** AML, Answer Set Programming, Boolean network, Proteomics data

## Abstract

**Background:**

During the last years, several approaches were applied on biomedical data to detect disease specific proteins and genes in order to better target drugs. It was shown that statistical and machine learning based methods use mainly clinical data and improve later their results by adding omics data. This work proposes a new method to discriminate the response of Acute Myeloid Leukemia (AML) patients to treatment. The proposed approach uses proteomics data and prior regulatory knowledge in the form of networks to predict cancer treatment outcomes by finding out the different Boolean networks specific to each type of response to drugs. To show its effectiveness we evaluate our method on a dataset from the DREAM 9 challenge.

**Results:**

The results are encouraging and demonstrate the benefit of our approach to distinguish patient groups with different response to treatment. In particular each treatment response group is characterized by a predictive model in the form of a signaling Boolean network. This model describes regulatory mechanisms which are specific to each response group. The proteins in this model were selected from the complete dataset by imposing optimization constraints that maximize the difference in the logical response of the Boolean network associated to each group of patients given the omic dataset. This mechanistic and predictive model also allow us to classify new patients data into the two different patient response groups.

**Conclusions:**

We propose a new method to detect the most relevant proteins for understanding different patient responses upon treatments in order to better target drugs using a Prior Knowledge Network and proteomics data. The results are interesting and show the effectiveness of our method.

**Electronic supplementary material:**

The online version of this article (10.1186/s12859-018-2034-4) contains supplementary material, which is available to authorized users.

## Background

Only one quarter of Acute Myeloid Leukemia (AML) diagnosed patients survive beyond 5 years. It is therefore worth exploring how mathematical modeling may contribute on a shift towards a more personalized follow up treatment for AML diagnosed patients. On this context, a prediction of the treatment response of AML patients, solely based on proteomic data, may add valuable information and improve clinical decisions. In 2014 the DREAM 9 challenge was launched in order to predict the complete remission (CR) and primary resistant (PR) response to chemotherapy of 191 AML patients from their proteomics data (231 measured proteins) and from 40 clinical data [1]. In several studies analyzing AML data [1–4] it was found that proteomic data is less discriminant than clinical data to predict patients’ response. In the Dream 9 challenge all methods used in a first attempt clinical data to discriminate patients’ response, and in a second attempt the 2 best performing methods used proteomic data to improve their prediction accuracy. A small set of proteins was considered to have a significant impact: PIK3CA, GSKAB, PTEN and NPM1. In [5] the authors proposed a biomarker detection method for the Dream 9 challenge data, which combines a machine learning framework with prior knowledge concerning the evolutionary conservation of the selected biomarkers. In their work they agree with previous studies on the low discriminant power of proteomic data: only two discriminant features came from proteomic data (PIK3CA and GSK3) and the rest were taken from the bio-clinical data. In this work, we propose a method to answer to the DREAM 9 challenge by including as prior information signaling networks. Even if the task of compiling signaling networks may be considered time demanding, many publicly available resources containing regulatory information currently exist such as KEGG [6], Reactome [7, 8], Pathway Commons [9], OmniPath [10] and NDEX [11]. Some of these resources have available tools or Cytoscape [12] plug-ins to extract networks given a list of molecules, such as ReactomeFIViz [13] for Reactome, CyPath2 [14] for Pathway Commons and PyPath [15] for OmniPath. Therefore, in this work we aim to understand the impact of using a mathematical model built over a signaling network, automatically retrieved from the KEGG database, associating the measured proteins on the prediction of CR-PR classes of patients’ response.

Patients’ response classification is usually approached by methods that find statistically significant markers from the transcriptomic or proteomic data at hand. A classical method used for this is univariate and multivariate Cox proportional hazards analyses. Following such approach, several statistic [16, 17] and machine learning [18–20] methods conceived for significant features extraction have been applied to this problem. This was the case for most of the best performing methods in the Dream 9 challenge. More recent approaches include the notion of pathways in this drug detection problem [21]. Such methods allow identifying the regulatory mechanisms related to the best drug targets [22] and this mechanistical information is valuable to understand the disease and the complexity of drug targeting. We have introduced in [23] the *caspo* method, which learns BNs from phosphoproteomic multiple perturbation data by using Logic Programming. This framework allows us to retrieve families of logic models having the best fit to the experimental data from exhaustive searches over a large-scale prior signaling network. In this work we make use of *caspo*. Experimentally, however, multiple perturbation data needed for *caspo* is impossible to obtain for patients. For this reason we have introduced a logic programming based approach to select subsets of proteins in the form of multiple perturbation experiments from static proteomics measurements that can allow us to maximize the discrimination between the two response type patients.

Following a parallel path to other Dream 9 challenge approaches, in this work we focused mainly on the proteomics data ignoring clinical data. We make this choice to discover discriminating signaling mechanisms. Our results show that 34 proteins were significant to build discriminant logic models of both classes of patients. We obtained the mechanisms and Boolean gates that best explained both type of data. Interestingly, several proteins are key in these models. Despite having two common proteins (ERBB3 and IGF1R), the Boolean networks present different interconnections among different proteins in the case of models that explain a CR response (FN1, SMAD6, LEF1, ERBB3, IGF1R, MAPK9, STMN1, GAPDH) and those that explain a PR response (FN1, YAP1, STK11, ERBB3, IGF1R, CASP9, CASP3, BAK1, TSC2, PTGS2). The PIK3CA and PTEN proteins, also reported in the previously DREAM 9 challenge cited methods, were also discovered by our approach, as intermediate nodes within the Boolean models.

When compared to the Dream challenge 100 patients testing dataset, the accuracy of the learned BNs was of 42%; this accuracy improves to 55% when selecting only patients where the measurements had strong signals. The accuracy obtained for the CR class, 64.7% (72.2% for strong signals) was greater than the one obtained for the PR class, 18.3% (27.2% for strong signals). In [1] it was found the same difference in the accuracy reported for different patient response groups (median accuracy of 73% for CR and 42% for PR); however, in that study the authors used the 40 bioclinical variables and only 4 protein measurements without considering the signaling mechanisms that explain this difference.

## Method

**Fig. 1:**
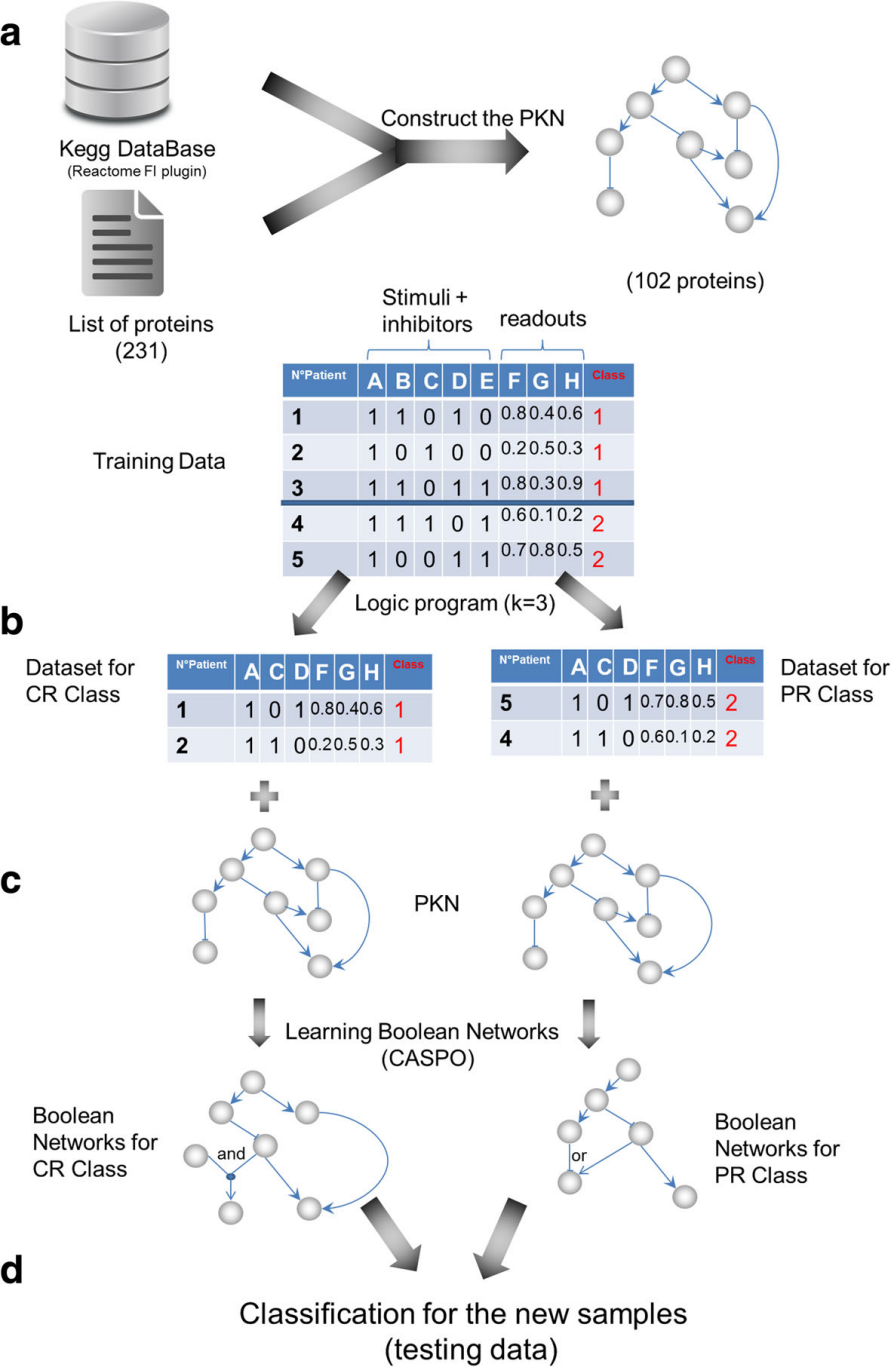
Workflow for our method. **a** PKN construction. In this step we pass the proteins present in our DREAM 9 dataset as input to the Cytoscape plug-in Reactome FI to construct the PKN. This plug-in finds all the paths between the input proteins across several databases, after that we select only relations coming from KEGG. **b** Protein and patient selection. This step consists on selecting *k* proteins from the dataset for which there is a maximum number of pairs of patients that have identical values in the *k* proteins but that belong to different response classes. **c** Learning. This step consists on finding the BNs for the two classes CR-PR corresponding to the two datasets obtained in step (**b**). (**d**) Classification. This step consists on classifying unknown patients datasets by using our learned logic models

Our method consists of four main steps. First, we start with the creation of a Prior Knowledge Network (PKN) from public databases that connects the 231 measured proteins. In this PKN we distinguished 3 types of nodes: stimuli, inhibitors and readouts. By stimuli we refer to the entry-layer of the network (nodes without predecessors); readouts, to the output-layer of the network (nodes without successors); and inhibitors, to proteins in between the entry and output-layers. The second step is the implementation of a logic program based on Answer Set Programming for proteins and patients selection. This logic program selects a group of *k* stimuli and inhibitor proteins that maximize the number of pairs of patients for which the binarized values of their experimental measures matched in both classes (CR, PR). In the third step we used the reduced dataset (composed of previously selected proteins and patients) to learn the Boolean networks (BNs) with the *caspo* software [24]. This step produces two families of BNs for the two response classes (CR and PR). Our objective here was to learn different families of BNs by using the identical stimuli-inhibitor cases and the maximal difference of readouts measures for each class and finally compare the structure and mechanisms between these BNs families. The final step is the classification step in which we compute the Mean Square Error (MSE) between measured readouts and predicted readouts for each patient in the testing data based on the two families of previously learned BNs. The given patient will be classified in the class with the lower MSE. The overall flowchart of our method is presented in Fig. 1. The different steps will be detailed in the following sections.

### Create the Prior Knowledge Network (PKN)

The first step of our workflow is to create the PKN from the proteomics dataset of the AML DREAM 9. DREAM challenges are crowd-sourcing challenges where biologists provide experimental data related to a particular and precise biological problematic and mathematicians, physicians and computer scientists develop methods to answer to the biological question.

**Data description.** The data consists of measurements of 191 patients diagnosed with AML who were treated at MD Anderson Cancer Center (USA). Each patient has 40 bio-clinical data measures and 231 protein levels measured using RPPA (Reversed Phase Protein Array). The patients are classified in two classes following their response to treatments: Complete Remission (CR), for patients with a good treatment response and Primary Resistant (PR), for patients with a bad treatment response.

**Network construction.** We construct the PKN by using the KEGG database [6] through Reactome FI [7, 25] which is a Cytoscape plug-in that queries several pathway databases, containing pathways and network patterns related to cancer and other types of diseases, such as Reactome and KEGG (see Fig. 1a). The plug-in connects the set of user-provided genes to build the PKN, representing each gene by a node, and each interaction between nodes by a signed arrow. We distinguish 3 types of nodes which are important for our modeling assumptions: stimuli, inhibitors, and readouts. Stimuli are nodes with no predecessors, readouts or measured nodes have no successors, and inhibitors, are nodes that connect stimuli to readouts. For our analyses we selected the KEGG’s interactions, since we found them semantically (expressing directionality and type of the interaction) more precise for our study than those in Reactome.

### Proteins and patients selection

The first step is data preprocessing. Since our modeling framework, *caspo*, aims to establish BNs that explain experimental measures, the proteomics data needs to be divided into *input* and *output* measures. *caspo* receives as input a list of perturbation data. Perturbation experiments are provided as Boolean values (stimulated or inhibited), while perturbation results are provided as continuous values in [0,1]. *caspo* will afterwards learn which BNs answer the input-output relation of the perturbation data. Therefore, for the input data (stimuli and inhibitor nodes), we discretized the proteomics data by using the k-means algorithm [26]. In this way we classified all the measurements into two clusters centered in {0,1} values; then we used the associated cluster for each value of the input data to select the class. See Eq. (1).

1 $$ \text{if}\ \left(\mathrm{1} - a_{ij} \right) \le 0.5\ \text{then}\ 1 \qquad\qquad \text{else}\ 0.  $$

we denote as *a*_*ij*_ the discretized value of the stimuli or inhibitor protein *i* for patient *j*. For the output data (readout nodes), we transformed each value of readout *i* for the patient *j* in a normalized value in the interval of [0,1] by using Eq. (2)

2 $$ r_{ij} = \frac{r^{\prime}_{ij}-{\min}}{{\max}-{\min}}  $$

where $r^{\prime }_{ij}$ is the non normalized value of the readout *i* for the patient *j* and *min* (resp. *max*) is the minimum (resp. maximum) value of all the readouts.

The second step is to conceive a logic program in Answer Set Programming (ASP) [27, 28] that given the proteomics data (see Fig. 1b) with discretized measures assigned to stimuli and inhibitors nodes, with normalized measures assigned to readout nodes, and where stimuli-inhibitor-readout preprocessed values were classified either into CR and PR patient classes, computes the following:

1. Select a set *K* of proteins, composed of *k* proteins from all combinations of stimuli and inhibitors $C_{|S|+|I|}^{k}$; where *S* and *I* represent the set of stimuli and inhibitor nodes respectively.
2. Select pairs of patients for which the discretized values of the proteins in *K* match in both classes (CR and PR).
3. Maximize the number of pairs of patients belonging to different classes. See optimization (3).
4. Maximize the difference in the readouts (proteins in *R*) of the pairs of patients selected. See optimization (4).

3 $$ \begin{aligned} & {\text{maximize}} & & \sum_{\text{j,j}^{\prime} \in \text{CR} \times \text{PR}}\mathrm{f}^{\mathrm{K}}(\text{j,j}^{\prime}) \\ & \text{subject to} & & f^{K}(j,j^{\prime})= 1 \; \text{if} \; a_{ij} = a_{i,j^{\prime}} \forall i \in K \\ &&& f^{K}(j,j^{\prime})= 0 \; \text{else}. \end{aligned}  $$

4 $$ \begin{aligned} & \underset{k}{\text{maximize}} & & \sum_{(\text{j,j}^{\prime}) \in \mathrm{A}_{\mathrm{k}}} \sum_{\mathrm{i}=1}^{|\mathrm{R}|} \left| \mathrm{r}_{\mathrm{i}}^{\text{CR}^{\mathrm{j}}} - \mathrm{r}_{\mathrm{i}}^{\text{PR}^{\mathrm{j}^{\prime}}} \right| \\ & \text{subject to} & & A_{k}: \text{one optimal set of} (\text{j,j}^{\prime}) \text{pairs} \\ &&& \text{selection of Step 3}. \end{aligned}  $$

From steps 1–3, the *k* proteins selection should maximize the number of CR vs. PR cases in which their discretized measures was identical. Step 4 is applied in the case where multiple optimal selections of patients’ pairs are proposed in Step 3. For example if we have more than one patient in the CR class that matches one or more patients in the PR class. In this case we choose the pair of patients that maximizes the difference of the readout nodes selected with the maximal CR vs. PR cases.

After presenting the general scheme of our method, now we provide details on its implementation in Answer Set Programming (ASP). The declarative approaches such as ASP are very suitable for selecting features that can differentiate the patients response and obtaining an efficient enumeration of solutions by a solver.

### Proteins and patients selection - ASP implementation

In this section we provide an overview of the ASP program used for protein selection.



In line 1, we represent the proteins *V* as facts over the predicate *node/1*, namely node(v) for all *v ∈ V*/ *V* are the nodes present in the PKN. In line 2, we represent the two classes of patients *C1* for the class Complete Remission and *C2* for th class Primary Resistant as facts using the predicate *class/1*. In lines 3–4, we represent *pert(E,V,S,C)* to say that the perturbation (experience or patient) number *(E)* for the protein *(V)*, is clamped to *S*, *S ∈ {0,1}*, and it belongs to the class *C*.



In line 1, we generate a set of k proteins with the predicate *selprot/1*, from all the proteins present in the perturbations. In fact this predicate generates all the possible ways to select *k* proteins from *D*, where *D* is the set containing all proteins of the DREAM 9 dataset. In line 2, we define the predicate *aff/4* that expresses that the perturbation *(E)* for the protein *(V)* in selprot/1, is clamped to *S*, *S ∈ {0,1}*, and it belongs to the class *C*. In line 3, we select the pairs of perturbations that have the same values in *S(S1=S2)*, but belong to different classes of patients *C*1<*C*2. *egale(I,J,V)* expresses that the perturbation *I* and the perturbation *J* belong to different classes and have the same value at the protein *V*. In line 4, we count the number of proteins where the perturbations *I* and *J* are equal, *i.e.*, we count the number of predicates *egale(I,J,V)*. If the number of proteins equal to *k* (selected above in line 1), then we can say that there is an affinity between experience *I* and experience *J*, *i.e.* they are similar on all *k* selected proteins. We represent that by the predicate *affinity/1* as shown in the line 5. Finally, in line 6, we maximize the number of *affinity/2*, *i.e.* the number of cases where I and J are similar and then we display the proteins *(selprot/1)* and affinities *(affinity/2)* found in lines 7–8.

For the sake of clarity we present the ASP code of the maximization of the readouts difference in the Additional file 1.

### Learning

The result of the logic program are 2 reduced datasets in the form of a matrix with the selected *k* proteins and optimal number of patients. These 2 datasets have the same number of patients, the same values of the *(k)* stimuli and inhibitor proteins, and different readout values. Each dataset belongs to either the CR or PR class. From these two files and the PKN we learned a family of Boolean Networks (BNs) with *caspo* for each class of patients (see Fig. 1c). *caspo* is a Python and ASP software to learn Boolean Networks (BN) from multiple samples data and a PKN [24].

### Classification

In order to predict the response to drugs for new patients from our logic models, we proposed 2 validation approaches (see Fig. 1d).

#### Method 1

Given a dataset associated to a new patient, we predict the value of the readout proteins in the new patient dataset from the two families of BNs learned in the previous section and from the binarized values of the stimuli and inhibitor proteins in this new dataset. Afterwards, we computed the Mean Square Error (MSE) between the BNs prediction and the readout measurements. We classified the patients to the class which had the lowest MSE.

#### Method 2

This method may give an *unknown* answer to the classification problem for some patients datasets. New patients datasets are only considered for classification if the normalized value *v* of their readouts proteins has a significant measure (*v*<0.25 or *v*>0.6). If the patient is kept, then we classify the patient according to Method 1.

Instead of predicting a binary value (complete response to therapy and achievement of remission or resistance to treatment), our method reports a value in [0,1] expressing the confidence that a patient will have a complete response and achieve complete remission. A predicted value of 1 indicates complete confidence that the patient will respond well to therapy and achieve complete remission. A predicted value of 0 indicates a complete confidence that the patient’s case will be resistant to treatment.

## Results

### Prior knowledge network

**Fig. 2:**
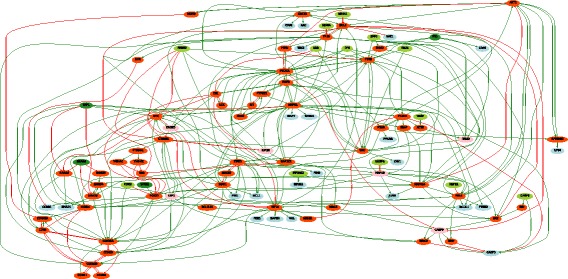
The Prior Knowledge Network is constructed from the 102 proteins in the DREAM 9 challenge proteomics dataset that are documented in the KEGG database. The dark- and light-green nodes are stimuli (nodes without predecessor), blue nodes are readouts (nodes without successors) and the red and pink nodes are inhibitors (rely the stimuli to readout nodes). Green arrows mean activation, red ones mean inhibition

We constructed a PKN from the KEGG database as explained in the “Method” section. We input the list of 231 proteins and selected the associations obtained from KEGG database only, without selecting linker proteins. The output is a PKN that has 102 nodes (17 stimuli, 62 inhibitors and 23 readouts) connected by 294 edges (see Fig. 2).

### Protein and patient selection

The result of this step was a subset of *k* proteins extracted from the union of the stimuli and inhibitors present in the PKN (79 proteins). Our logic program was parametrized to the value *k*, which choice was arbitrary. This value has impact on the following BN learning step. Choosing a larger *k* will allow us to build larger networks and therefore larger logic models; however it may also imply less patient couples (experimental conditions) to learn and therefore less data-specific models.

**Fig. 3:**
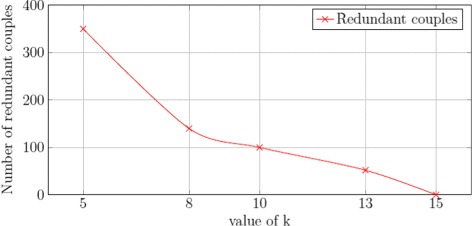
Impact of *k* on the number of redundant-couples. A redundant-couple is one that includes a patient that is associated in another couple. This chart represents how the number of redundant-couples evolves with respect to the number of proteins selected (*k*)

**Fig. 4:**
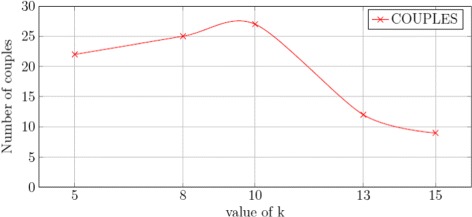
Impact of *k* on the number of couples of patients selected. This chart represents how the number of couples of patients evolves with respect to the number of proteins selected (*k*)

To choose the best value for *k* we run our algorithm of protein selection with different values. For each selected *k* we compared the numbers of couples of patients (experimental conditions) obtained. In this analysis we deleted the redundant couples by using the readout maximization described in the “Method” section and the Additional file 1. Since a couple is defined as a patient-to-patient association, it may happen that different couples associate the same patient, we name such couples as *redundant couples*. We plot the number of redundant couples with respect to different values of *k* and we observed (see Fig. 3) that this number decreases rapidly when *k* increases. Our logic program maximizes the number of non-redundant couples. The maximum number of non-redundant couples is plotted in Fig. 4. We observed that the highest value of non-redundant couples is obtained when choosing *k*=10. From this analysis, we chose *k*=10 to keep a good compromise between the total number of couples explored and the choice of the non-redundant ones.

We therefore selected 10 proteins from the set of all stimuli and inhibitors combinations ($C_{79}^{10} $). The total number of patients was reduced to two subsets corresponding to the two classes of patients (CR, PR) of size 26 (see Additional file 2). These reduced datasets were composed of 4 stimuli, 6 inhibitors (see dark-green and red nodes in Fig. 2) and 23 readouts for 26 different patients in each class. Notice that the values of the stimuli and inhibitors were shared, while the readout values differed. There were in total 52 different patients. In this case, given that we only obtained one result that maximized the number of non-redundant patient couples for *k*=10, we did not use the readout maximization (see Eq. 4).

We evaluated the effect of filtering the original protein dataset (231 proteins) by using 2 feature selection methods, based on network clustering and principal component analyses, that selected the best proteins distinguishing both response classes. We found (see Additional file 3) that the number of proteins was reduced respectively to 69 and 58. However, once this subset of proteins was given to the ASP protein-patient selection logic program, the number of maximized patients was of 21 for both. This number of patients was lower than 26, and therefore such possibility was excluded from our analysis in order to build more data-specific models.

### Learned Boolean networks

We learned the two families of BNs (CR vs PR) using the *caspo* software providing as input data the same PKN (see Fig. 2) and the 2 reduced datasets (matrix of 33 proteins by 26 patients) for each CR/PR class. In Table 1 we describe the case-studies and the learned BNs. All of our computational tests were performed using clingo 4, and a computation facility, *Bird platform* [29], with 320 nodes and 1.3To RAM. In this table we show the number of nodes and edges for each PKN and the number of possible BNs derived from the PKN that will be explored by *caspo* exhaustively. We restrict the search space for BNs to hyperedges with up to 2 source nodes, which yields logical networks having AND gates with up to 2 inputs. *caspo* learned a family of optimal BNs for each CR/PR class. The CR family had 10 BNs, while the PR one had 9. The size (number of logic clauses) of the optimal BNs for the CR case was of 24, while it was of 29 in the PR BNs. The Mean Square Error (MSE) between the respective datasets and the optimal BNs are slightly equal (≈0.112). After learning the boolean networks, the *caspo* classify function, analyzes the networks and groups them according to their input-output behaviors. For the CR family we got one behavior and for the PR family we got 2 behaviors, this points to more mechanisms in the PR case.

**Table 1:** Description of the case study

Cases studies						Learn					Classify	
Case	Nodes	Edges	Search space	Perturbations	Readouts	MSE	Size	Networks	*t*_*learn*_	*t*_*opt*_	I/O	*t*_*I*/*O*_
CR	102	294	2^834^	26	23	0.1123	24	10	6339	4779	1	1
PR	102	294	2^834^	26	23	0.1120	29	9	1588	3654	2	1

PKN and dataset for both CR-PR classes. The column *Search space* describes how many BNs, derived from the PKN, were explored by *caspo*. The column *Perturbations* refers to the different couples of patients selected by our algorithm. The column *learn* outputs *caspo* results in terms of optimal BNs learned description. *MSE* shows the BN fitness (Mean Square Error with respect to the dataset), *size* the number of logic clauses of the BN, *Networks* the number of optimal BNs found. The column *t*_*learn*_ is the learning time, while the *t*_*opt*_ column shows the optimization time in minutes. The column *classify* shows an analysis of the BNs learned. *I/O* shows the number of different logic behaviors and *t*_*I*/*O*_ the computation time in minutes

**Fig. 5:**
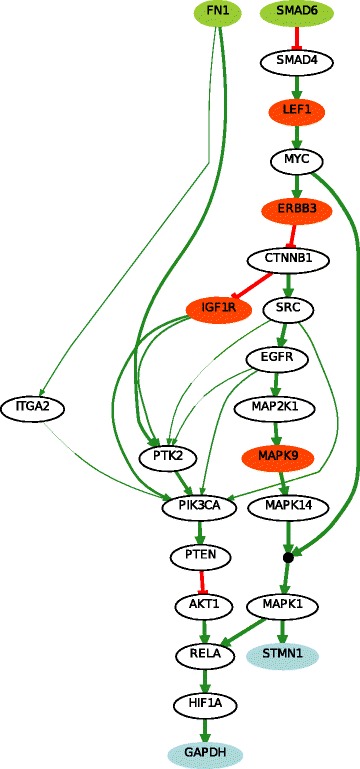
Boolean network of CR class. This figure represents the union of optimal BNs learned from the initial PKN and the reduced patients dataset from CR class. This BN can explain and predict the measurements of readouts STMN1 and GAPDH starting from the stimuli FN1 and SMAD6, passing by the inhibitors LEF1, ERBB3, IGF1R and MAPK9, and other intermediate proteins. The thicker edges represent those that are the most frequent paths in the BN family. The association between a node and its predecessors is an AND gate if it is preceded by a filled black circle and an OR gate otherwise

**Fig. 6:**
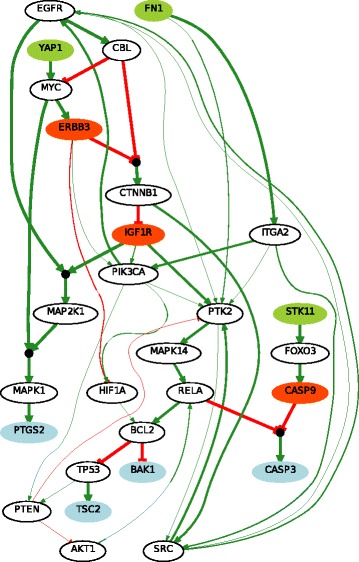
Boolean network of PR class. This figure represents the union of optimal BNs learned from the initial PKN and the reduced patients dataset from PR class. This BN explains and predicts the measurement of readouts PTGS2, TSC2, BAK1 and CASP3 starting from the stimuli FN1, YAP1 and STK11, passing by the inhibitors ERBB3, IGF1R and CASP9, and other intermediate proteins. The thicker edges represent those that are the most frequent paths in the BN family. The association between a node and its predecessors is an AND gate if it is preceded by a filled black circle and an OR gate otherwise

**Fig. 7:**
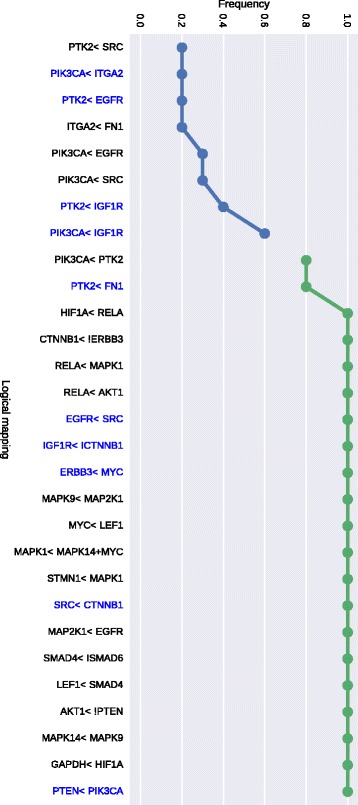
Mapping frequency for CR class. Blue clauses or logical maps are common to both CR and PR patient response groups

**Fig. 8:**
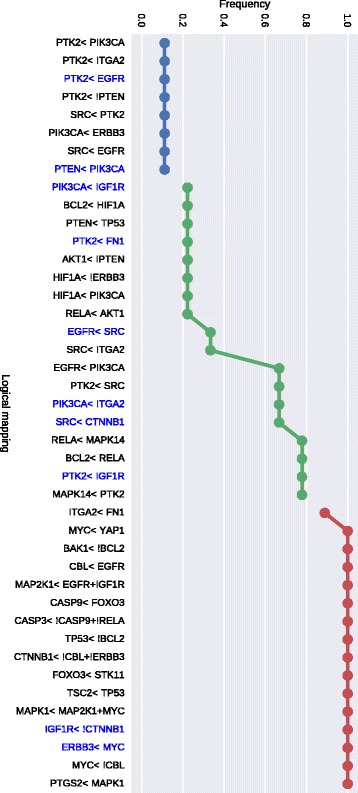
Mapping frequency for PR class. Blue clauses or logical maps are common to both CR and PR patient response groups

In Figs. 5 and 6 we illustrate the union of the BNs retrieved for the CR and PR case respectively. The two families of BNs are different and explain different behaviors. Interestingly they do not connect the same subset of stimuli, inhibitors and readouts. The common stimulus in both cases is FN1, the common inhibitors (or intermediate nodes) are ERBB3 and IGF1R, and there is not a common readout present in both families. This may show that while the perturbation values of stimuli and inhibitor nodes were the same, the difference in the readout values create this variability; showing a more complex structure associated to patients models with a primary resistant (PR) response. This is also shown in Table 1 in the larger number of logic mechanisms (I/O) that can be obtained in the BNs of PR class. In order to analyze the logic clauses in both families we plot in Figs. 7 and 8 the frequency of the logic clauses. The frequency in [0,1] of a logic clause measures its presence across all BNs in the family. We computed 10 logic clauses that appear in both, CR and PR, families (blue clauses in Figs. 7 and 8). These common mechanisms represent the 34% of the total clauses in the CR class and the 23% of the total clauses in PR. More than 50% of these common logic mechanisms are having a frequency higher than 0.6 within their respective families. We also observe that the majority (72%) of logic clauses in CR are highly redundant (frequency >0.6). This redundancy feature is less present in the PR class, since only 57% of the logic clauses have a frequency higher than 0.6. These last figures enhance the observation that the PR model contains different and less redundant logic mechanisms compared to the CR model.

### Validation results

We applied both classification methods described in the “Method” section to the learning (52 patients) dataset, which corresponds to the reduced version of the 191 patients used to learn the BNs families; and to the testing dataset (100 patients) provided by the DREAM 9 challenge. We summarized these results in Table 2.

**Table 2:** Description of the accuracy results with the two classification methods using the Boolean network predictions

	Learning dataset			Testing dataset		
Method	Accuracy CR	Accuracy PR	Balanced accuracy	Accuracy CR	Accuracy PR	Balanced accuracy
Method 1	57.6% (26)	53.8% (26)	55.7% (52)	64.7% (72)	18% (28)	41.35% (100)
Method 2	80% (10)	37.5% (8)	58.75% (18)	72.2% (18)	27.2% (11)	49.7% (29)

The accuracy was computed for the learning dataset of 52 patients, and the testing dataset of 100 patients. The numbers in parenthesis correspond to the number of patients that were analyzed

The accuracy rate for learning and testing datasets differs. In previous results (Table 1) we show that the two families of BNs could similarly predict the data with a MSE of 0.11. However, the MSE is not fully related to the accuracy of each model in predicting the correct patient response class. Our BNs proposed an accuracy of 52% for the learning dataset (57.6% for CR and 53.8% for PR cases). This accuracy improves to 61% (80% for CR and 37.5% for PR cases) when filtering our learning dataset to patients with significant readout values, that is, by choosing readout signals closer to Boolean behaviors. In Table 2 we show this improvement in the Method 2 row. We can see that this improvement in accuracy costs a reduction of the patients that can be classified. For the learning dataset 34 patients could not be classified.

For the testing data, the predictions from the learned BNs proposed a balanced accuracy (BAC) of 41,35%. The accuracy of the CR class is higher (64,7%) when compared to the PR class (18,3%). In [1] it was found the same difference in the median accuracy for the different patient response groups (73% for CR and 42% for PR) accross all DREAM 9 participating methods. However, DREAM 9 participating methods used mainly bioclinical variables to extract the model features and at most 2 protein measurements. One of the reasons of this difference is that the number of CR and PR samples differ, this can be seen in Table 2 in the values in parenthesis which represent the number of patients analyzed. When using the classification method after filtering patients with not significant readout values, we found a BAC of 49,75%. The accuracy for the CR class was of 72,2%, while of 27,2% for the PR class. In Additional file 4 we show a table summarizing the comparison of the BAC and AUROC (area under the receiver operating characteristic) scores obtained with our method, compared to the two first-ranked methods in the DREAM 9 challenge as well as with respect to the median of the 31 participating methods. In this table we show the number and nature of the features used to build the classification models for the compared methods. We can notice that our method is the one that uses the largest number of protein features: 30 protein features and 71 logical rules relating the behavior among proteins in our Boolean models. This characteristic allows deriving mechanistic models, which require a sufficient number of protein information. The fact of not considering clinical data, penalizes our BAC and AUROC scores. Interestingly, for the CR class, our accuracy remains comparable to other methods of the challenge.

## Discussion and conclusion

The DREAM challenge dataset is a large proteomics dataset that may contain noise in some of its measurements. Including all dataset proteins in a predictive model may lead to over-fitting and pre-selecting a subset of proteins add bias as well. We validated for this case-study the last assumption (see Additional file 5 for details). On a first attempt we tried to build Boolean networks from a subset of 20 significant (top ranked p-value after applying Student test between PR and CR patients) proteins. However the accuracy of such learned models was poor (22%) and both of the BNs families (CR and PR) learned had the same logic behaviors. These preliminary results inspired us to develop a mathematical framework to select (*k*) proteins that distinguish these two families by imposing several constraints, such as *maximize the number of patient samples that have the same values of some proteins (so-called stimuli and inhibitors) where these samples belong to different classes (CR or PR)*. Such dataset proposed the same measurements over the same input-nodes and different measurements over outputs-nodes across the different (CR/PR) classes and allowed us to build response-specific logic models. The different connections among CR vs. PR logic models could stand for mutations in the cell population systems that usually appear after exposure to chemotherapies. The logic models obtained in this study validate this hypothesis because we found that the logic mechanisms of resistant patients were more varied that those of complete remission ones.

Given a large dataset, our method detects the most relevant proteins to build predictive models in order to distinguish two classes of patients. These models could be trained with larger datasets and used to represent the mechanisms within disease models to better target drugs. In this work, we discovered a family of logic models that discriminate the response of Acute Myeloid Leukemia (AML) patients to treatment. The protein selection logic program was implemented using Answer Set Programming. This method allowed us to build a reduced dataset. Later, *caspo* allowed us to train BNs from a Prior Knowledge Network to this reduced dataset. From this analysis, we obtained two BNs (CR vs. PR) families. These models allow us to classify new patient datasets in patient response groups. Our results, evaluated on the AML testing data from DREAM challenge 9, show that we obtain different topologies with similar and divergent logic mechanisms for each type of patient response group. The accuracy of such models is low compared to the DREAM 9 challenge methods, mainly because we did not include the clinical data. Nevertheless, for CR patients our models had an accuracy of 64.7% using only proteomics data; and this accuracy improved to 72.2% when restricting the classification to patients with significant readout measurements. We believe that the low trend in PR accuracy, also observed in [1], is due in part to the small number of PR cases compared to the CR cases in the testing data (28 PR vs. 72 CR). Interestingly, this low accuracy trend in PR cases, applies as well to the learning dataset, which evidences the fact that the learned BNs predict better CR than PR cases, specially in cases of patients with significant measured readouts where the accuracy difference was 80% vs. 37.5% in CR compared to PR cases. Differently from other methods participating in the DREAM 9 challenge, our method is able to propose precise mechanistic explanations of the difference among the two patient response groups in the form of Boolean models.

In a continuation of this work, we aim to provide a better understanding of the patient classification accuracy rate, which was not the main scope of our paper. We believe this question deserves further attention because the learned BNs models show that in this dataset some proteins seem more relevant than others, and that observing a normalized protein value close to 1 or 0 is more significant for the learning step. Also, using other cancer diseases patient datasets can be a challenging test for our method.

## Additional files

Additional file 1 ASP implementation for readouts maximization. A short description of asp coding for selecting couples that have the same values of inputs. (PDF 37 kb)

Additional file 2 Dataset reduction. This figure illustrates the dataset reduction, starting with a huge dataset and getting two small datasets to use later on in the learning step. (PDF 700 kb)

Additional file 3 Feature selection methods. This table show the techniques explored for feature selection. (PDF 45 kb)

Additional file 4 A comparison between our method and the results obtained by the DREAM 9 challenge participants. This table present a comparison of our method and the results obtained by DREAM 9 challenge participants. (PDF 48 kb)

Additional file 5 Learning Boolean Networks from a statistically selected subset of proteins. This figure section present previous works to learning BNs from statistically selected subset of proteins. (PDF 41 kb)
