# Supplementary material for: Discriminate the response of Acute Myeloid Leukemia patients to treatment by using proteomics data and Answer Set Programming
Source: BMC Bioinformatics. 2018 Mar 8;19(Suppl 2):59. doi: 10.1186/s12859-018-2034-4 (PMC5850944; doi:10.1186/s12859-018-2034-4)
Supplement: Supplementary file 1 — ASP implementation for readouts maximization. A short description of asp coding for selecting couples that have the same values of inputs. (PDF 37 kb) [file 12859_2018_2034_MOESM1_ESM.pdf]

Discriminate the response of Acute Myeloid Leukemia patients to treatment by using Proteomics Data and Answer Set Programming

Lokmane Chebouba, Bertrand Miannay, Dalila Boughaci and Carito Guziolowski

Additional file 1 : ASP implementation for readouts maximization

1- Dataset representation for readouts maximization

prot(Z).

affinite(L1,L2).

r(L1,Z,X).

We represent the set of readouts nodes with the the predicate prot/1 namely prot(Z)

for all the readouts nodes.

The predicate affinity(L1,L2) is the result of the program in the Listing 2 (Methods

Section - Protein Selection – main paper). It means that the patient L1 and patient

L2 have the same values for the k choosen proteins.

r(L1,Z,X) means that the value of the readout Z for the patient L1 is X.

2- ASP Implementation of the maximization of the readouts

1.diff(L1,L2,Z,D) :- affinite(L1,L2), r(L1,Z,X), r(L2,Z,Y), gene(Z), D=X-Y,

X>Y.

2.diff(L1,L2,Z,D) :- affinite(L1,L2), r(L1,Z,X), r(L2,Z,Y), gene(Z), D=Y-X,

Y>X.

3.somme(L1,L2,M) :- M = #sum {D: diff(L1,L2,Z,D)}, affinite(L1,L2).

4.choix(L1,L2) :- {somme (L3,L2,M): M>X}0, {somme (L1,L3,M): M>X}0, somme

(L1,L2,X).

5.sommedsommes(S) :- S = #sum {M: somme(L1,L2,M)}.

6.#show somme/3.

7.#show choix/2.

8.#show sommedsommes/1.

In line 1 and line 2, we calculate the difference between the readouts for all pairs of patients over the predicate diff/4, namely diff(L1,L2,Z,D) which means that the difference between the patient L1 and the patient L2 is D for the readout node Z.

Then in line 3, we sum the difference between L1 and L2 for all the readouts nodes with the predicate somme/3 namely somme(L1,L2,M) which means that the sum of differences between L1 and L2 for all readouts is M.

In line 4, we choose the patients that have the largest difference in the readouts vector between patients L1 and L2, with the predicate choix/2 namely choix(L1,L2).

In line 5, if we had more than one answer set that maximize the differences, we choose the one that maximize the sum of diferences.

Line 6,7 and 8 display the results in the terminal.
