# Supplementary material for: Discriminate the response of Acute Myeloid Leukemia patients to treatment by using proteomics data and Answer Set Programming
Source: BMC Bioinformatics. 2018 Mar 8;19(Suppl 2):59. doi: 10.1186/s12859-018-2034-4 (PMC5850944; doi:10.1186/s12859-018-2034-4)

Discriminate the response of Acute Myeloid Leukemia patients to treatment by using Proteomics Data and Answer Set Programming

Lokmane Chebouba, Bertrand Miannay, Dalila Boughaci and Carito Guziolowski

Additional file 2 : Dataset reduction

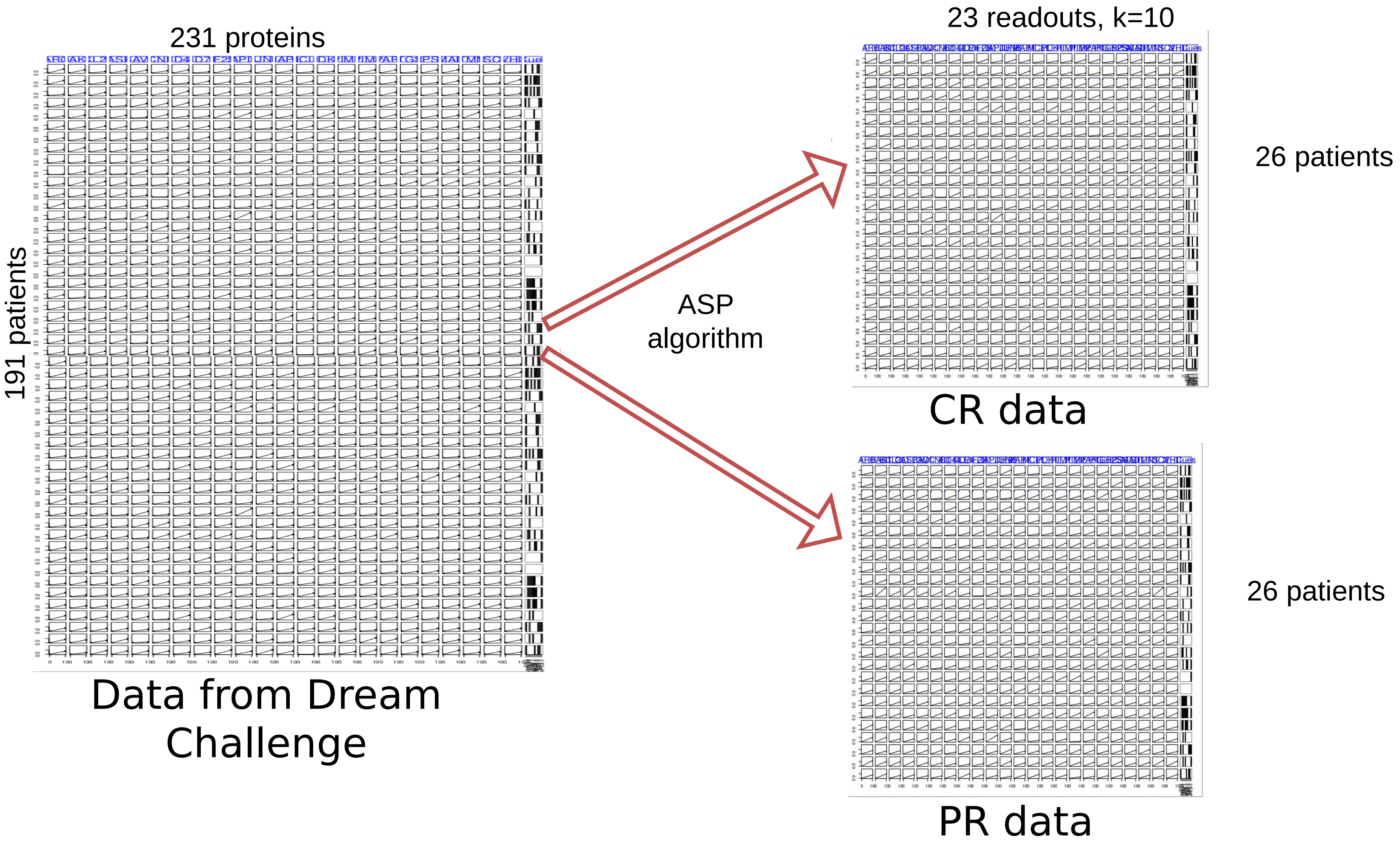

Supplement: Supplementary file 2 — Dataset reduction. This figure illustrates the dataset reduction, starting with a huge dataset and getting two small datasets to use later on in the learning step. (PDF 700 kb) [file 12859_2018_2034_MOESM2_ESM.pdf]
