# Supplementary material for: Discriminate the response of Acute Myeloid Leukemia patients to treatment by using proteomics data and Answer Set Programming
Source: BMC Bioinformatics. 2018 Mar 8;19(Suppl 2):59. doi: 10.1186/s12859-018-2034-4 (PMC5850944; doi:10.1186/s12859-018-2034-4)
Supplement: Supplementary file 3 — Feature selection methods. This table show the techniques explored for feature selection. (PDF 45 kb) [file 12859_2018_2034_MOESM3_ESM.pdf]

Discriminate the response of Acute Myeloid Leukemia patients to treatment by using Proteomics Data and Answer Set Programming

Lokmane Chebouba, Bertrand Miannay, Dalila Boughaci and Carito Guziolowski

Additional file 3 : A comparison between our method and the winners of the DREAM 9 challenge

We applied 2 feature selection methods (one supervised and other unsupervised) in order to filter the original set of proteins (231) to a more meaningful set, as proposed. This protein subset will be given then to the ASP feature-patient selection method. The first method used a Cytoscape plugin called CytoNCA [1] to rank the top 70% of features using the betweenness centrality, the other one used a Principal Component attribute selector with WEKA [2,3]. Then we applied our ASP feature-patient selection method. We obtained a number of pairs of patients which was lower than 26 in both techniques as shown in the table below.

Feature selection method	number of features selected	number of pairs of patients
Best 70 % features	69	21
Principal component	58	21

Having a low number of patients that can be distinguished among both, CR and PR, response classes using the proteomic, implies that the size of the Boolean networks distinguishing these patient sets will be small and with less power to differentiate the CR and PR mechanisms. We maintain that adding the Prior Knowledge Networks with the protein-patient ASP implemented selection allow us to restrict the original protein domain to 10 proteins, for which the number of patients distinguishing both classes is maximized (26). This number is impossible to obtain when the domain of proteins is reduced a priori with the feature selection methods studied.

References :

[1] Yu Tang, Min Li, Jianxin Wang, Yi Pan, Fang-Xiang Wu, CytoNCA: A cytoscape plugin for centrality analysis and evaluation of protein interaction networks, In Biosystems, Volume 127, 2015,

Pages 67-72, ISSN 0303-2647, https://doi.org/10.1016/j.biosystems.2014.11.005.

[2] Eibe Frank, Mark A. Hall, and Ian H. Witten (2016). The WEKA Workbench. Online Appendix for "Data Mining: Practical Machine Learning Tools and Techniques", Morgan Kaufmann, Fourth Edition, 2016.

[3] Mark Hall, Eibe Frank, Geoffrey Holmes, Bernhard Pfahringer, Peter Reutemann, and Ian H. Witten (2009). The WEKA Data Mining Software: An Update. SIGKDD Explorations, Volume 11, Issue 1.
