# Supplementary material for: Discriminate the response of Acute Myeloid Leukemia patients to treatment by using proteomics data and Answer Set Programming
Source: BMC Bioinformatics. 2018 Mar 8;19(Suppl 2):59. doi: 10.1186/s12859-018-2034-4 (PMC5850944; doi:10.1186/s12859-018-2034-4)
Supplement: Supplementary file 4 — A comparison between our method and the results obtained by the DREAM 9 challenge participants. This table present a comparison of our method and the results obtained by DREAM 9 challenge participants. (PDF 48 kb) [file 12859_2018_2034_MOESM4_ESM.pdf]

Discriminate the response of Acute Myeloid Leukemia patients to treatment by using Proteomics Data and Answer Set Programming

Lokmane Chebouba, Bertrand Miannay, Dalila Boughaci and Carito Guziolowski

Additional file 4 : A comparison between our method and the results obtained by the DREAM 9 challenge participants

In this Table we sumarize the classification scores when compared to the methods participating in the DREAM 9 challenge. Our method is the only one using 33 protein variables included in a mechanistic Boolean model, which can predict the output of some proteins according to a different scenario. The Boolean network families obtained by our method for each patient response class have in total 71 logical rules. Methods 1 and 2 refer to our classification methods without and with filtered readouts (see the Classification subsection of the Methods section in the main paper). We can see that DREAM 9 participating methods included at most 2 protein features in their analysis. Our method doesn't include clinical data. This fact penalizes the BAC and AUROC classification score of our method. However it predicts with a comparable accuracy to most of the DREAM participating methods the Complete Remission patients. The BAC and AUROC scores were computed using the same DREAM 9 formulas specified at https://www.synapse.org/#!Synapse:syn2455683/wiki/64624

	**PKN + Boolean Model + ASP (method 1)**	**PKN + Boolean Model + ASP (method 2)**	**DREAM 1st ranked (Evolutionary model & machine learning)**	**DREAM 2nd ranked (machine learning: regression)**	**DREAM median (across 31 participating teams)**
**Discriminating Data (#features)**	Proteomic (33) + BNs (71 clauses)	Proteomic (33) + BNs (71 clauses)	Clinical + Proteomic + Evolutionary model (30 features, including 2 proteins)	Clinical (3)	Clinical + Proteomic (<40 clinical and <4 proteins)
**CR**	64.7%	72.2%	74%	68%	73%
**PR**	18%	27.2%	81%	76%	42%
**BAC**	41.35%	49.75%	77.9%	72.8%	57.5%
**AUROC**	0.74	0.63	0.79	0.78	0.64
