# Supplementary material for: Discriminate the response of Acute Myeloid Leukemia patients to treatment by using proteomics data and Answer Set Programming
Source: BMC Bioinformatics. 2018 Mar 8;19(Suppl 2):59. doi: 10.1186/s12859-018-2034-4 (PMC5850944; doi:10.1186/s12859-018-2034-4)
Supplement: Supplementary file 5 — Learning Boolean Networks from a statistically selected subset of proteins. This figure section present previous works to learning BNs from statistically selected subset of proteins. (PDF 41 kb) [file 12859_2018_2034_MOESM5_ESM.pdf]

Discriminate the response of Acute Myeloid Leukemia patients to treatment by using Proteomics Data and Answer Set Programming

Lokmane Chebouba, Bertrand Miannay, Dalila Boughaci and Carito Guziolowski

Additional file 5 : Learning Boolean Networks from a statistically selected subset of proteins

We used the NCI-PID database to generate a PKN. For this purpose, we associated for each protein the p-value based on the Student test between PR and CR patients.

Then, we selected the top 20 proteins and generated a subgraph from NCI-PID connecting those proteins to each other by the shortest paths.

Based on this subgraph containing 154 nodes and 177 edges and the Dream 9 challenge dataset, we learned with caspo two sets of BNs for PR and CR patients.

Then, we computed the MSE for each patient and each set of BNs.

Based on this computing, we were able to compare the two MSE for each patient.

We can notice that only 37.5% of patients had two different MSE with BNs from PR et CR data.

Moreover, only 22% of the patients had a smaller MSE with the class they belong to while the remaining patients (15.5%) had a smaller MSE with the other class.

Due to those results, we can conclude that this predictive model has an accuracy of 22% and is not able to efficiently discriminate PR and CR patients.
